# New distribution records for the rare genus *Afrotremex* Pasteels (Siricidae: Hymenoptera) and provision of interactive Lucid identification keys to species

**DOI:** 10.3897/BDJ.3.e7160

**Published:** 2015-11-23

**Authors:** Simon van Noort, Henri Goulet

**Affiliations:** ‡ Natural History Department, Iziko South African Museum, Cape Town, South Africa; § Biological Sciences Department, University of Cape Town, Cape Town, South Africa; | Agriculture and Agri-Food Canada, Ottawa, Canada

**Keywords:** *
Afrotremex
*, Afrotropical region, Africa, distribution, endemic, Hymenoptera, identification keys, Lucid matrix key, Siricidae, Siricoidea

## Abstract

**Background:**

*Afrotremex* Pasteels, 1951 is a rare genus of wasps endemic to the Afrotropical region, containing 6 species represented by 14 specimens. Specimens were previously only recorded from central Africa: Cameroon, Congo, Democratic Republic of Congo, Gabon and Uganda.

**New information:**

Here we record two additional specimens housed in the Natural History Museum in London (BMNH), one of which is a male of *A. xylophagus* Goulet, 2014 collected in Ghana (previously Gold Coast). This record extends the known distribution of the genus into west Africa, and represents the second known male specimen for the genus. The other BMNH specimen is a female paratype of *A. violaceus* Pasteels, 1951 collected in the Democratic Republic of Congo. We provide high quality photographs of these additional two specimens. Images of all six known species are openly available online on WaspWeb. In addition we have developed interactive online Lucid Matrix and Lucid Phoenix identification keys to the species, which are openly available on WaspWeb at: http://www.waspweb.org/Siricoidea/Siricidae/Keys/index.htm

## Introduction

*AfrotremexPasteels 1951* is the only indigenous genus representative of Siricidae in the Afrotropical region (Goulet 2014, van Noort 2015). The other siricid genus present in the region is represented by the alien pest species, *Sirex noctilio* Fabricius, 1793, accidentally introduced to South Africa (Hurley et al. 2012, Taylor 1962, Tribe 1995, Tribe and Cillié 2004, van Noort and Picker 2011) from the western Palaeartic region on several independent occasions via Oceania and South America (Boissin et al. 2012). The recent revision of *Afrotremex* elevated the known species richness from two to six, with the description of four new species, based on 12 specimens examined in the collections of the Hungarian Natural History Museum, Budapest, Hungary (HNHM); Musée Royal de l’Afrique Centrale, Tervuren, Belgium (MRAC); National Museum of Natural History, Smithsonian Institution, Washington, DC, USA (USNM); Museum für Naturkunde der Humboldt Universität, Berlin, Germany (ZMHB) (Goulet 2014). This is a rare genus of wasps endemic to Africa that is poorly represented in world collections. The genus was previously only recorded from central Africa: Cameroon, Congo, Democratic Republic of Congo, Gabon and Uganda (Goulet 2014). Subsequently, two additional specimens were located in the collections of the Natural History Museum in London. We have photographed these additional two specimens and provide these images here as well as on WaspWeb. Images of all six known species were published in Goulet (2014) and are available online on WaspWeb (van Noort 2015). In addition we have developed interactive online Lucid Matrix and Lucid Phoenix identification keys to the species, based on the dichotomous keys published in Goulet (2014).

## Materials and methods

### Photography

Images were acquired using the Leica LAS 4.4 multi-stacking imaging system. The Leica system comprised a Leica® Z16 microscope and a Leica DFC450 Camera. Leica Application Suite V 4.4 software was used to manage image acquisition using an automated Z-stepper and merging of the image series into a single in-focus image. Images were post-processed in Adobe Photoshop and plates produced using Adobe illustrator. Specimens were imaged using the EntoVision multiple-focus imaging system to illustrate diagnostic characters.

### Lucid identification key production

Online interactive keys were produced using Lucid 3.5 Builder and Lucid Phoenix Builder meeting the requirements of publishing both static and dynamic interactive keys under an open access model (Penev et al. 2009). Character matrices were generated and edited within the Lucid 3.5 Builder environment as input into the interactive Lucid matrix key production (Penev et al. 2009). Interactive dichotomous keys were developed within the Lucid Phoenix builder environment. All keys were illustrated using high quality annotated images, highlighting diagnostic characters. The images are integrated into the key above each couplet resulting in a user-friendly output. This key format circumvents the requirement of familiarity with morphological terminology associated with a particular taxonomic group, because the characters are visually illustrated and annotated making the keys usable by a wide range of end-users including the non-specialist.

### List of depositories

BMNH: The Natural History Museum, London. Curators David Notton & Gavin Broad.

## Data resources

### Online identification keys

A static dichotomous identification key to species of *Afrotremex* was published in (Goulet 2014). We have developed interactive online Lucid Matrix and Lucid Phoenix identification keys to the species, based on the identification key published in Goulet (2014). These keys are available on WaspWeb at: http://www.waspweb.org/Siricoidea/Siricidae/Keys/index.htm. End users can choose between three different key formats depending on their personal preference. Although Lucid Phoenix keys are interactive keys they are still dichotomous and a choice needs to be made at each key couplet to continue. Lucid matrix keys, on the other hand, use a different approach where relevant states from multiple character features can be selected independently until identification is achieved. For more information concerning Lucid keys visit http://www.lucidcentral.org.

## Taxon treatments

### Afrotremex violaceus

Pasteels, 1951

#### Materials

- **Type status:** Paratype. **Occurrence:** recordedBy: Rev. P. Van Eyen; individualCount: 1; sex: female; lifeStage: adult; preparations: pinned; disposition: damaged; **Taxon:** scientificName: *Afrotremex violaceus* Pasteels, 1951; kingdom: Animalia; phylum: Arthropoda; class: Insecta; order: Hymenoptera; family: Siricidae; genus: Afrotremex; specificEpithet: violaceus; scientificNameAuthorship: Pasteels, 1951; taxonomicStatus: valid; **Location:** country: Democratic Republic of Congo; locality: Mayidi; verbatimLocality: Mayidi; **Identification:** identifiedBy: J. Pasteels; Henri Goulet; dateIdentified: 1951; 2015; **Event:** eventDate: 1942; year: 1942; verbatimEventDate: 1942; **Record Level:** modified: 10/17/2015; language: en; collectionID: BMNH; institutionCode: BMNH; basisOfRecord: PreservedSpecimen

#### Description

See Goulet 2014. Photographs of the additional paratype female are provided in Figs 1, 2.

#### Distribution

Democratic Republic of Congo

#### Biology

Unknown.

### Afrotremex xylophagus

Goulet, 2014

#### Materials

- **Type status:** Other material. **Occurrence:** recordedBy: W.H. Patterson; individualCount: 1; sex: male; lifeStage: adult; preparations: pinned; disposition: damaged; **Taxon:** scientificName: *Afrotremex xylophagus* Goulet, 2014; kingdom: Animalia; phylum: Arthropoda; class: Insecta; order: Hymenoptera; family: Siricidae; genus: Afrotremex; specificEpithet: xylophagus; scientificNameAuthorship: Goulet, 2014; taxonomicStatus: valid; **Location:** country: Ghana; locality: Aburi; verbatimLocality: Aburi; **Identification:** identifiedBy: Henri Goulet; dateIdentified: 2015; **Event:** eventDate: 1912-1913; year: 1912; 1913; verbatimEventDate: 1912-13; **Record Level:** modified: 10/17/2015; language: en; collectionID: BMNH; institutionCode: BMNH; basisOfRecord: PreservedSpecimen

#### Description

See Goulet 2014. Photographs of the additional male specimen are provided in Figs 3, 4.

#### Distribution

Cameroon, Ghana

#### Biology

Unknown.

## Checklists

### Checklist of *Afrotremex* species

#### Afrotremex comatus

Goulet, 2014

http://www.waspweb.org/Siricoidea/Siricidae/Afrotremex/Afrotremex_comatus.htm

##### Distribution

Uganda

#### Afrotremex hyalinatus

(Mocsáry, 1891)

http://www.waspweb.org/Siricoidea/Siricidae/Afrotremex/Afrotremex_hyalinatus.htm

##### Distribution

Congo; Gabon

#### Afrotremex opacus

Goulet, 2014

http://www.waspweb.org/Siricoidea/Siricidae/Afrotremex/Afrotremex_opacus.htm

##### Distribution

Democratic Republic of Congo

#### Afrotremex pallipennis

Goulet, 2014

http://www.waspweb.org/Siricoidea/Siricidae/Afrotremex/Afrotremex_pallipennis.htm

##### Distribution

Democratic Republic of Congo

#### Afrotremex violaceus

Pasteels, 1951

http://www.waspweb.org/Siricoidea/Siricidae/Afrotremex/Afrotremex_violaceus.htm

##### Distribution

Democratic Republic of Congo

#### Afrotremex xylophagus

Goulet, 2014

http://www.waspweb.org/Siricoidea/Siricidae/Afrotremex/Afrotremex_xylophagus.htm

##### Distribution

Cameroon, Ghana

## Discussion

Of the two additional specimens located in the collections of the Natural History Museum in London (BMNH), one is a male of *A. xylophagus* Goulet, 2014 collected in Ghana (previously Gold Coast). This record extends the known distribution of the genus and this species into west Africa. This is only the second male specimen known for the genus. The other BMNH specimen is a female paratype of *A. violaceus* Pasteels, 1951 from the Democratic Republic of Congo. Both specimens are damaged with missing wings, legs and antenna. The head and pronotum of both specimens have, at some point, been glued back onto the rest of the body.

The life history and biology of *Afrotremex* species is poorly known. The two ZMBH specimens of *A. xylophagus* were reared from a common widespread central African tree species, *Antrocaryon klaineanum* Pierre (Anacardiaceae), and since species of the related genera *Tremex* and *Eriotremex* are recorded as utilizing a range of angiosperm trees as hosts (Schiff et al. 2012) it is possible that *Afrotremex* are not necessarily highly host specific either (Goulet 2014). The genus may be genuinely rare, but it is more likely that the preferred habitat, probably the forest canopy (Goulet 2014), has not been adequately sampled. Deploying canopy sampling techniques such as fogging or elevated Malaise traps and yellow pan traps may facilitate acquisition of more material. Use of emergence traps or siting Malaise and yellow pan traps over, or around, fallen logs will likely also increase success.

## Supplementary Material

XML Treatment for Afrotremex violaceus

XML Treatment for Afrotremex xylophagus

XML Treatment for Afrotremex comatus

XML Treatment for Afrotremex hyalinatus

XML Treatment for Afrotremex opacus

XML Treatment for Afrotremex pallipennis

XML Treatment for Afrotremex violaceus

XML Treatment for Afrotremex xylophagus

## Figures and Tables

**Figure 1a.**
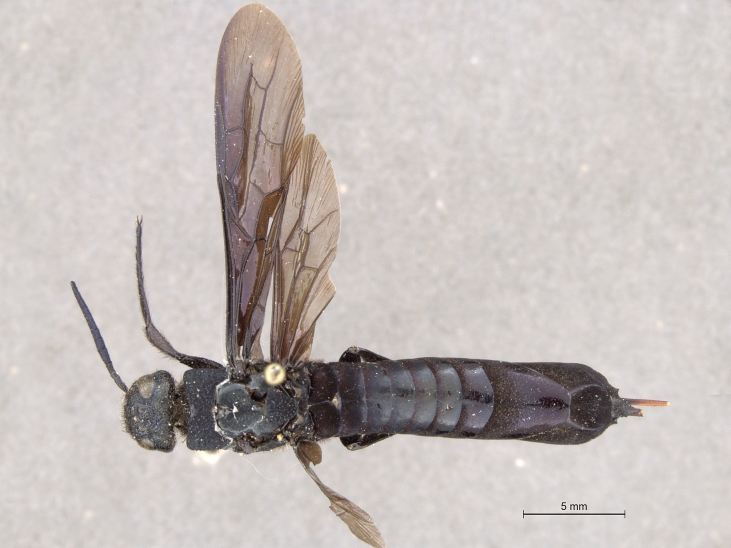
habitus, dorsal view.

**Figure 1b.**
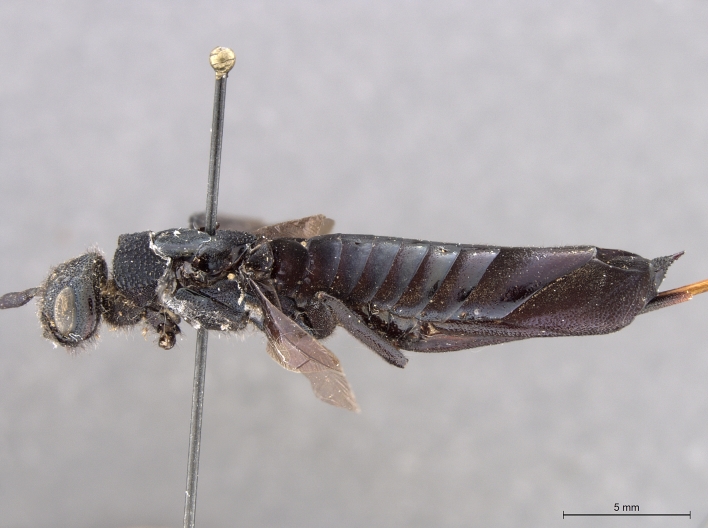
habitus, lateral view.

**Figure 1c.**
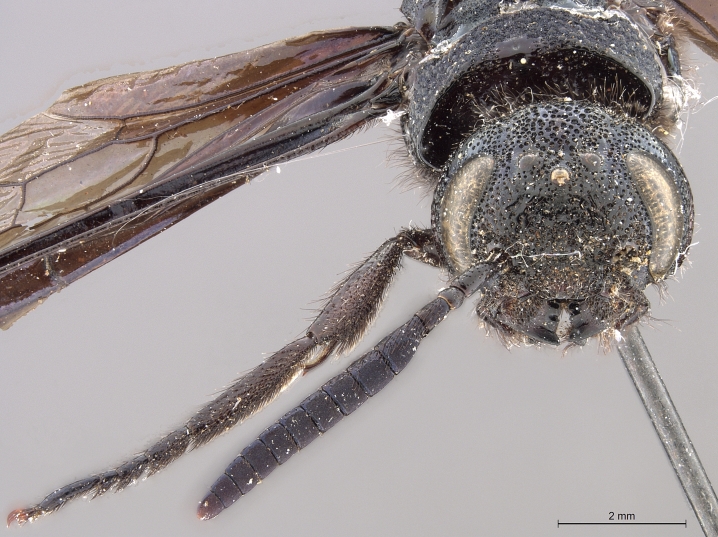
head, antenna anterior view.

**Figure 1d.**
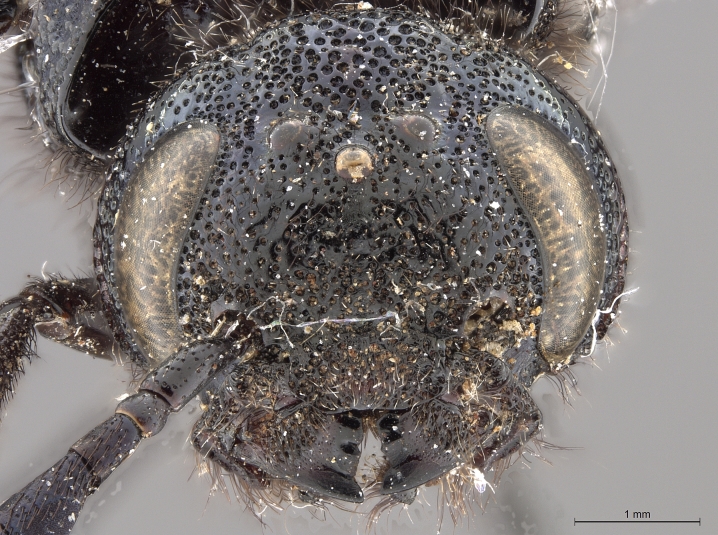
head, anterior view.

**Figure 1e.**
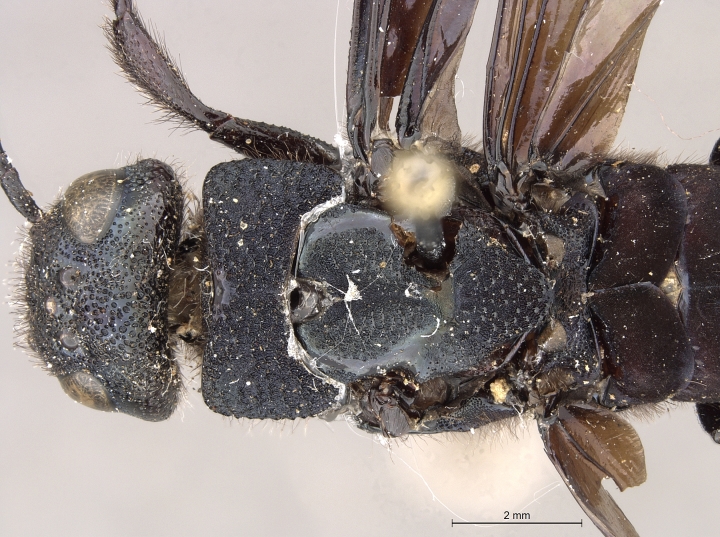
head, thorax, dorsal view.

**Figure 1f.**
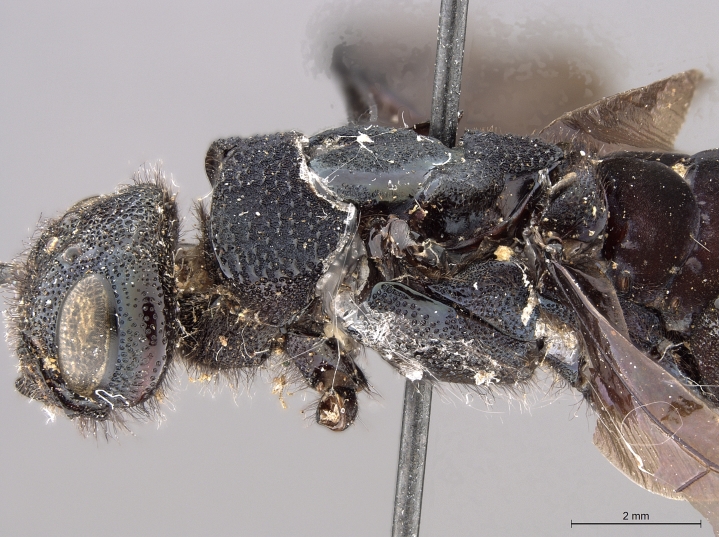
head, thorax, lateral view.

**Figure 2a.**
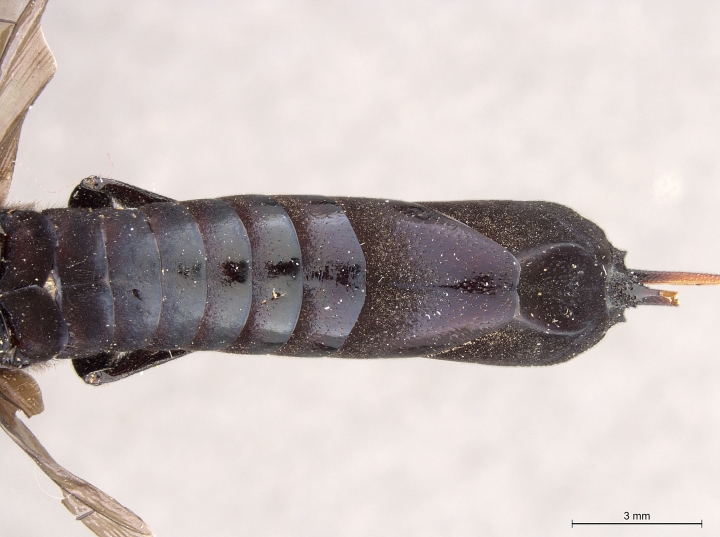
abdomen, dorsal view.

**Figure 2b.**
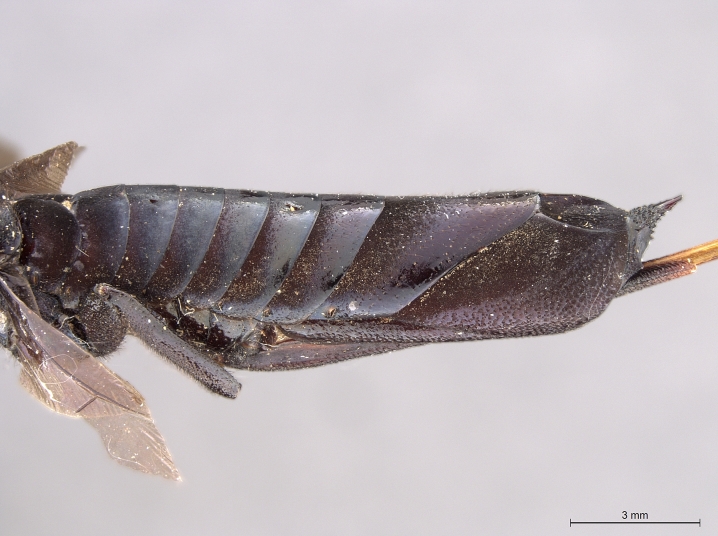
abdomen, lateral view.

**Figure 2c.**
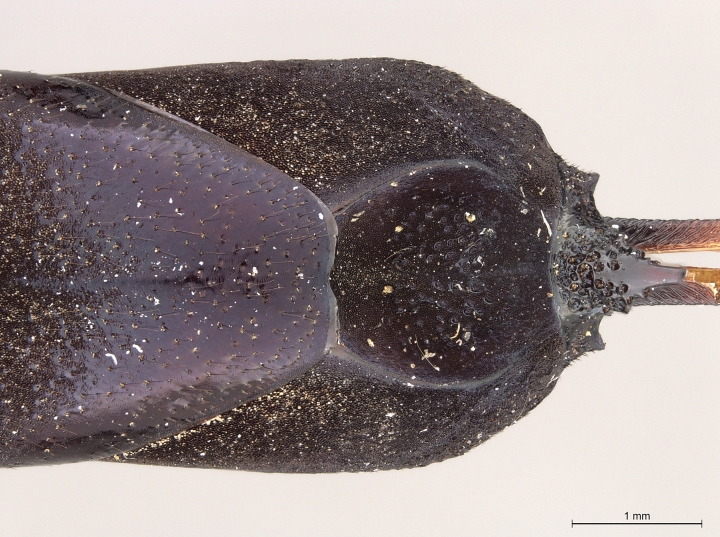
terminal abdominal segments, dorsal view.

**Figure 2d.**
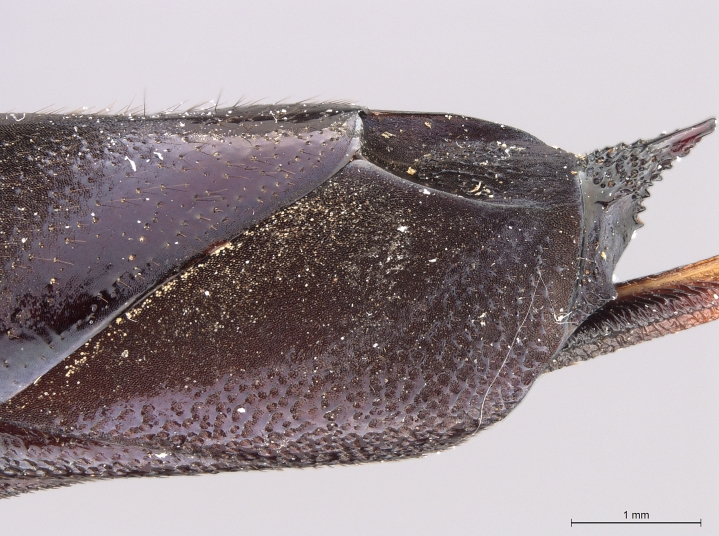
terminal abdominal segments, lateral view.

**Figure 2e.**
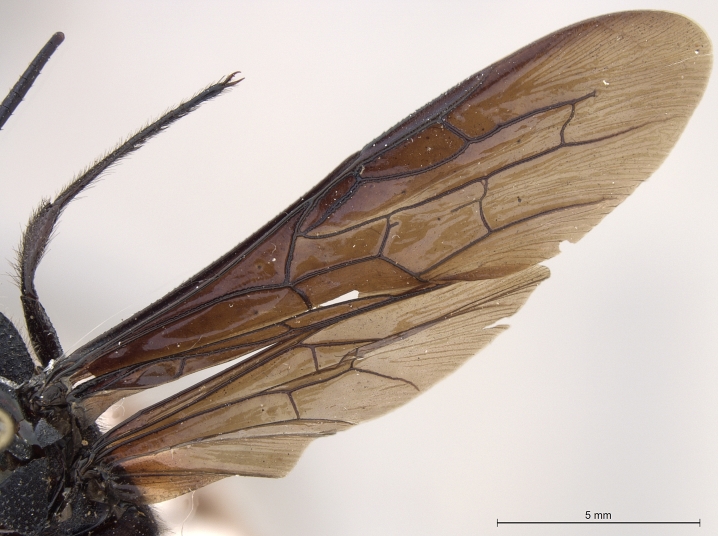
forewing and hind wing, dorsal view.

**Figure 2f.**
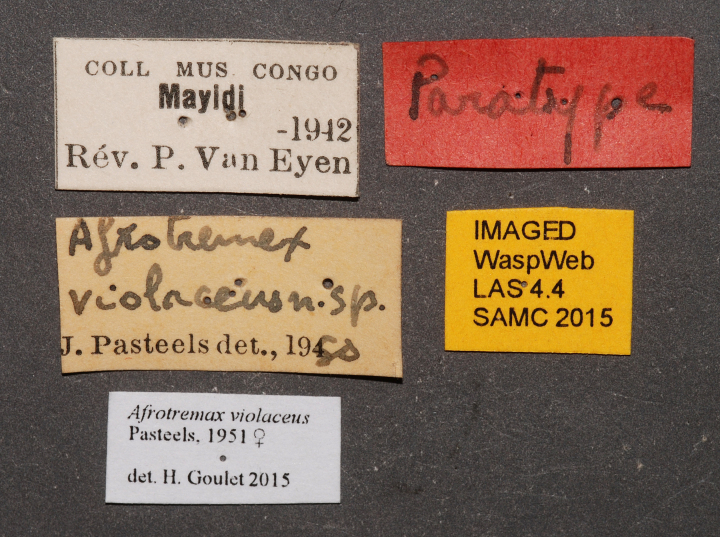
specimen data labels.

**Figure 3a.**
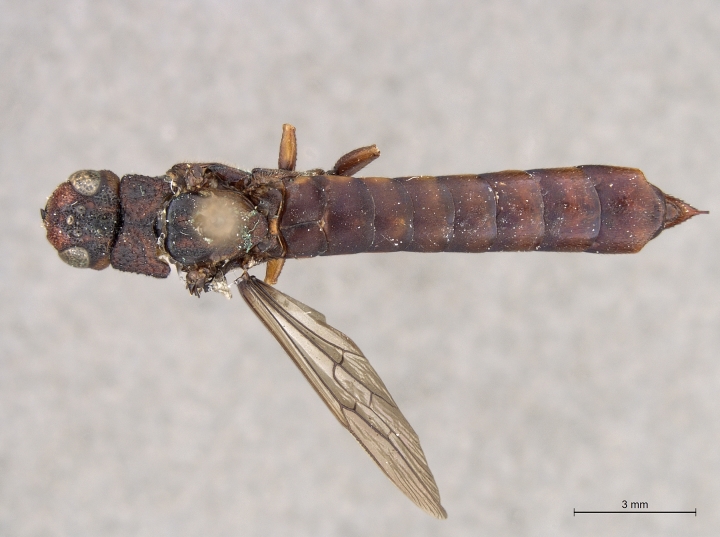
habitus, dorsal view.

**Figure 3b.**
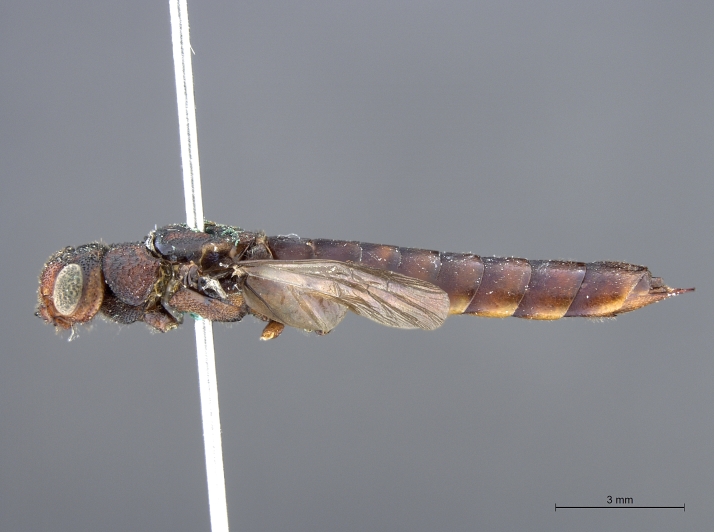
habitus, lateral view.

**Figure 3c.**
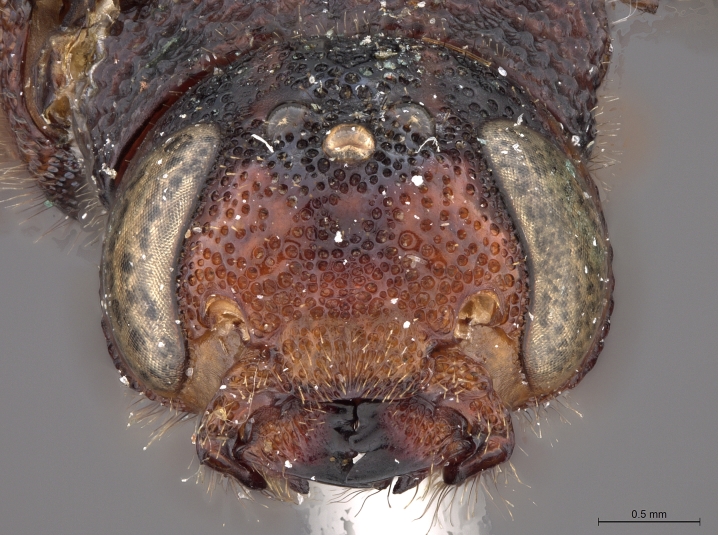
head, ventro-anterior view.

**Figure 3d.**
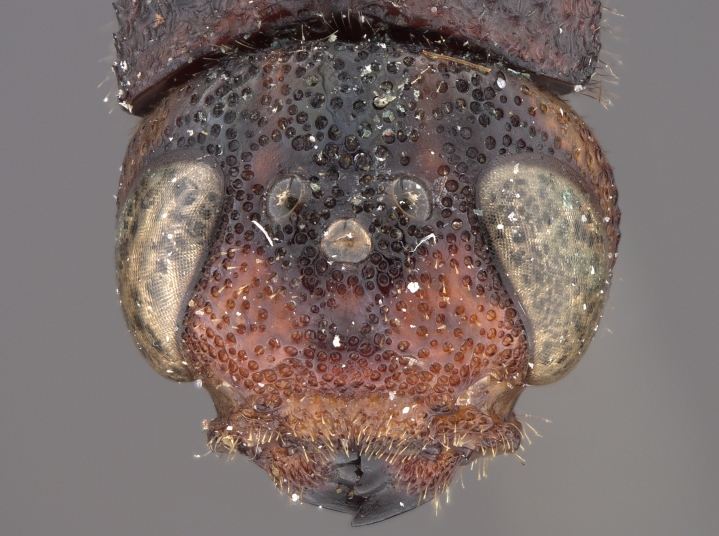
head, anterior view.

**Figure 3e.**
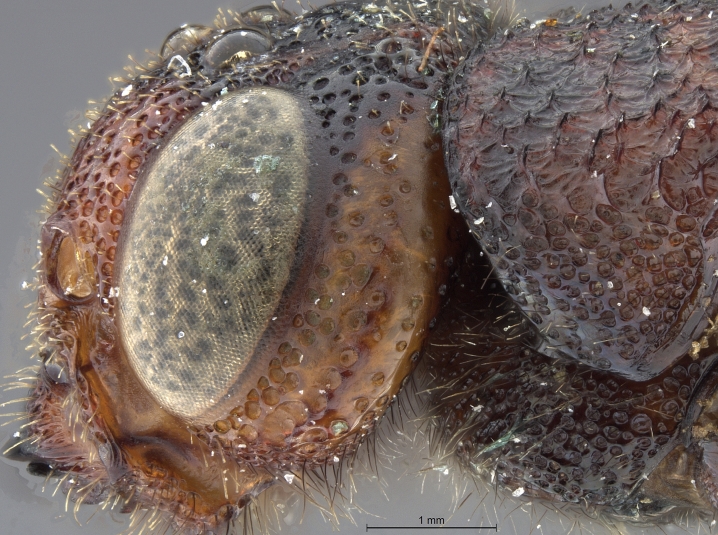
head, pronotum, lateral view.

**Figure 3f.**
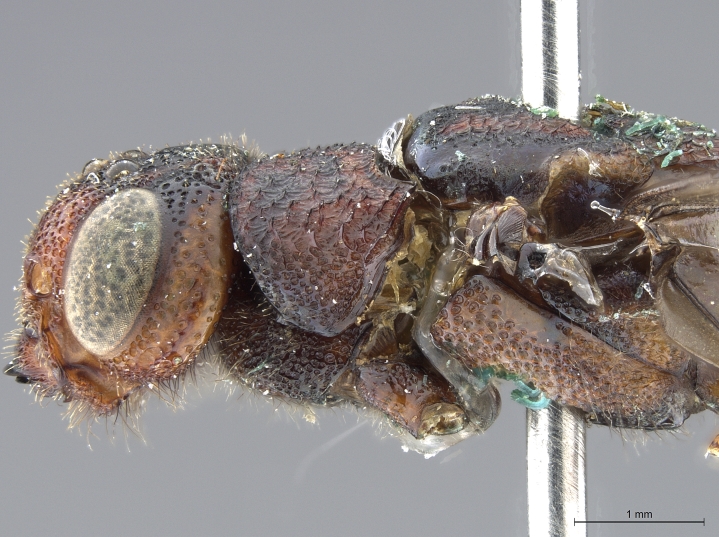
head, thorax, lateral view.

**Figure 4a.**
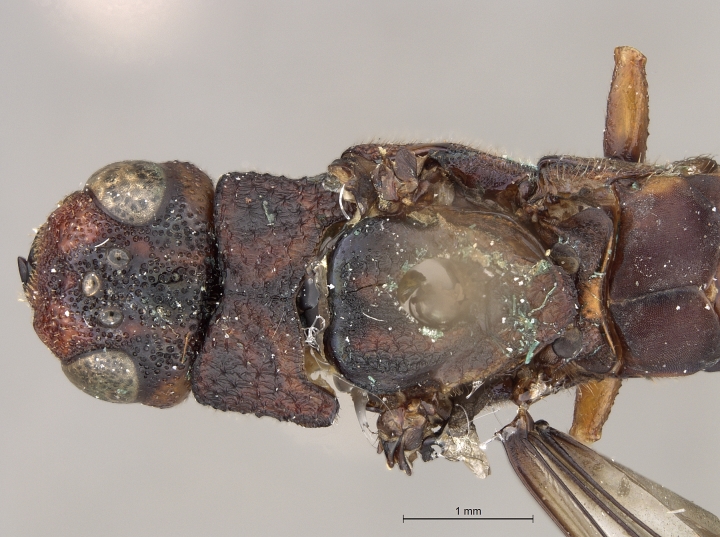
head and thorax, dorsal view.

**Figure 4b.**
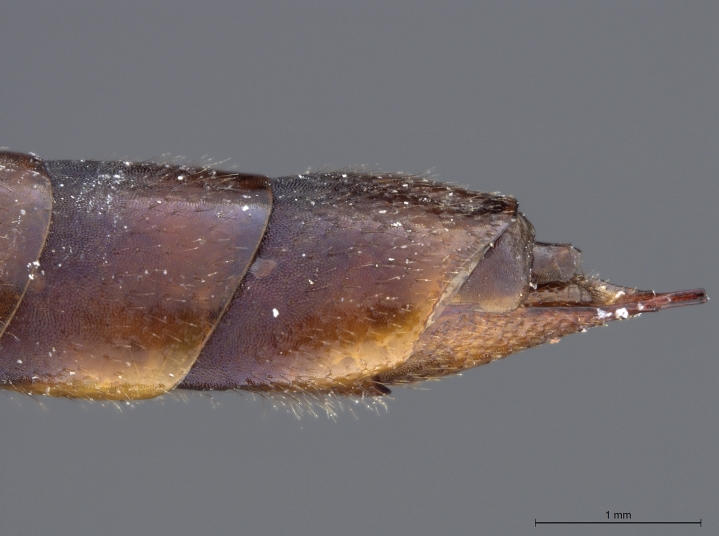
terminal abdominal segments, lateral view.

**Figure 4c.**
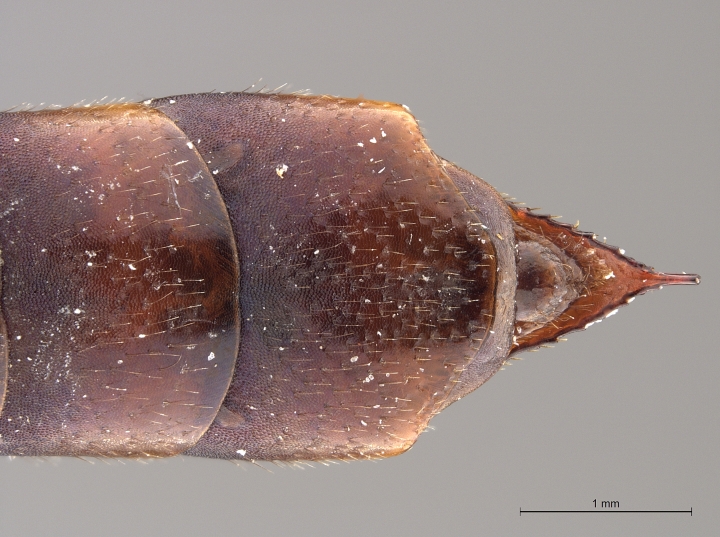
terminal abdominal segments, dorsal view.

**Figure 4d.**
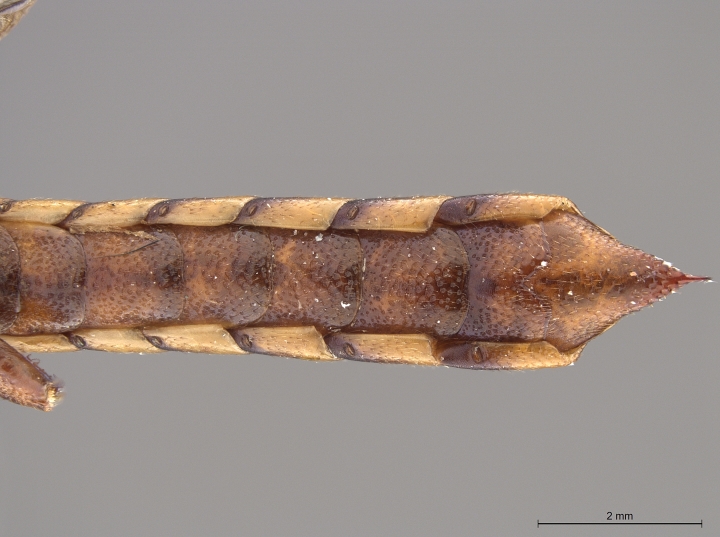
abdomen, ventral view.

**Figure 4e.**
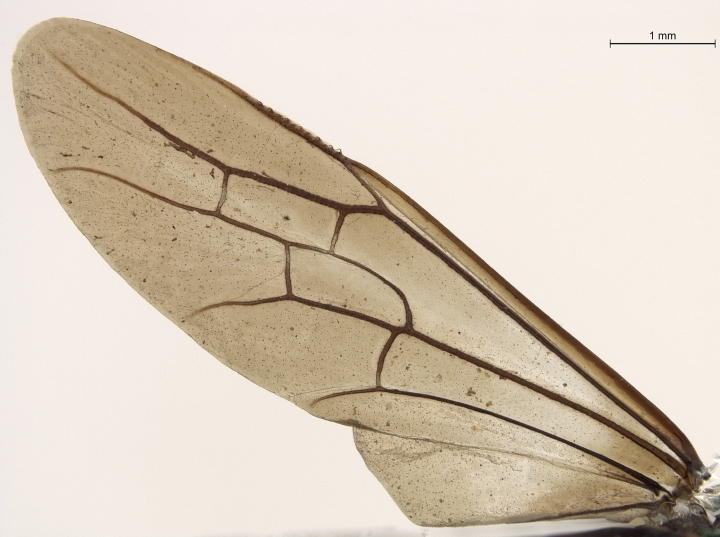
forewing, dorsal view.

**Figure 4f.**
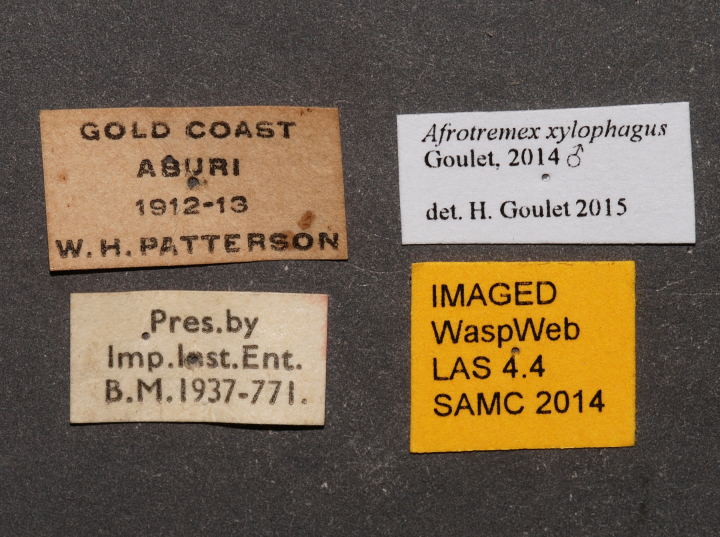
specimen data labels.
